# Disposition of Cannabidiol Metabolites in Serum and Urine from Healthy Individuals Treated with Pharmaceutical Preparations of Medical Cannabis

**DOI:** 10.3390/ph13120459

**Published:** 2020-12-12

**Authors:** Ana Pilar Pérez-Acevedo, Francesco Paolo Busardò, Roberta Pacifici, Giulio Mannocchi, Massimo Gottardi, Lourdes Poyatos, Esther Papaseit, Clara Pérez-Mañá, Soraya Martin, Annagiulia Di Trana, Simona Pichini, Magí Farré

**Affiliations:** 1 Clinical Pharmacology Unit, Hospital Universitari Germans Trias i Pujol and Institut de Recerca Germans Trias i Pujol (HUGTiP-IGTP), 08916 Badalona, Spain; appereza.germanstrias@gencat.cat (A.P.P.-A.); lpoyatos@igtp.cat (L.P.); epapaseit.germanstrias@gencat.cat (E.P.); cperezm.mn.ics@gencat.cat (C.P.-M.); smartins.mn.ics@gencat.cat (S.M.); mfarre.germanstrias@gencat.cat (M.F.); 2 Department of Pharmacology, Therapeutics and Toxicology, Universitat Autònoma de Barcelona, Cerdanyola del Vallés, 08193 Bellaterra, Spain; 3 Department of Excellence-Biomedical Sciences and Public Health, Università Politecnica delle Marche, 60121 Ancona, Italy; annagiulia.ditrana@gmail.com; 4 National Centre on Addiction and Doping, Istituto Superiore di Sanità, 00161 Rome, Italy; roberta.pacifici@iss.it (R.P.); simona.pichini@iss.it (S.P.); 5 School of Law, University of Camerino, 62032 Camerino, Italy; giulio.mannocchi@unicam.it; 6 Comedical srl, 38123 Trento, Italy; massimo.gottardi@comedical.biz

**Keywords:** cannabis, cannabidiol, cannabidiol metabolism, medical cannabis

## Abstract

The use of cannabis flowering tops with standardized amounts of active phytocannabinoids was recently authorized in several countries to treat several painful pathological conditions. The acute pharmacological effects and disposition of Δ-9-tetrahydrocannabinol (THC), cannabidiol (CBD), their acidic precursors and THC metabolites after oil and decoction administration have been already described. In this study, the disposition of CBD metabolites: 7-carboxy-cannabidiol (7-COOH-CBD), 7-hydroxycannabidiol (7-OH-CBD), 6-α-hydroxycannabidiol (6-α-OH-CBD), and 6-β-hydroxycannabidiol (6-β-OH-CBD) in the serum and urine of healthy volunteers was presented. Thirteen healthy volunteers were administered 100 mL of cannabis decoction in the first experimental session and, after 15 days of washout, 0.45 mL of oil. Serum and urine samples were collected at different time points, and the CBD metabolites were quantified by ultra-high-performance liquid chromatography–tandem mass spectrometry. The most abundant serum metabolite was 7-COOH-CBD, followed by 7-OH-CBD, 6-β-OH-CBD, and6-α-OH-CBD, after decoction and oil. Both 7-OH-CBD and the 6-α-OH-CBD showed similar pharmacokinetic properties following administration of both cannabis preparations, whereas 7-COOH and 6-α-OH-CBD displayed a significant higher bioavailability after decoction consumption. All CBD metabolites were similarly excreted after oil and decoction intake apart from 6-α-OH-CBD, which had a significantly lower excretion after oil administration. The pharmacokinetic characterization of CBD metabolites is crucial for clinical practice since the cannabis herbal preparations are increasingly used for several pathological conditions.

## 1. Introduction

Δ-9-tetrahydrocannabinol (THC) and cannabidiol (CBD) are the most abundant and pharmacologically active phytocannabinoids of the *Cannabis sativa* L. plant [1,2,3,4,5]. Whereas THC produces psychotropic and addictive effects, being a partial agonist at CB1 and CB2 receptors, CBD, as an antagonist, presents anxiolytic, myorelaxant, and antiepileptic properties without psychotropic or addictive effects [6,7,8].

Unlike recreational cannabis, which contains elevated proportions of THC, medical cannabis contains both THC and CBD at different proportions, together with terpenoids and other minor phytocannabinoids. It has been demonstrated that all these constituents are necessary to provide the best pharmacological effects for the treatment of chronic neurogenic pain, multiple sclerosis with muscle spasticity, amyotrophic lateral sclerosis, cachexia, cancer, glaucoma, Acquired immunodeficiency syndrome, Crohn disease, post-traumatic stress disorder, resistant epilepsy, and seizures [6,8].

Since cannabis decoction and oil are the most used medical cannabis herbal preparations, we recently investigated the disposition of THC, CBD, their acidic precursors (Δ-9-tetrahydrocannabinolic acid A-THCA-A and cannabidiolic acid—CBDA) and THC principal metabolites in the serum, oral fluid, sweat patch, and urine of healthy individuals treated with these two products [9].

Indeed, it was the first time that the pharmacokinetics of phytocannabinoids present in medical cannabis was explored in healthy subjects, since two previous studies involved children and young adults with drug-resistant epilepsy [10,11] and medication-overuse headache patients [12].

At that time, only THC metabolites could be detected and their kinetics investigated in our biological samples, while CBD metabolites could not be measured due to the unavailability of chemical standards. However, we believed that the examination of CBD metabolites should have been essential to better understand the mechanisms underlying the effects of different preparations of medical cannabis and of CDB-based pharmaceutical products.

Once CBD metabolites became commercially available, we investigated the disposition and pharmacokinetics of cannabidiol-7-oic acid (7-COOH-CBD), 7-hydroxycannabidiol (7-OH-CBD), 6-alpha-hydroxycannabidiol (6-α-OH-CBD), and 6-beta-hydroxycannabidiol (6-β-OH-CBD), in serum and urine samples stored from the previous study concerning healthy individuals treated with a single administration of cannabis decoction and cannabis oil containing similar amounts of phytocannabinoids.

## 2. Results

### 2.1. Subjects and Study Design

Fourteen subjects were initially recruited to complete the study. However, one male showed high serum concentration of THC metabolites at baseline time (0 h) and was therefore excluded from the study, so that finally, 13 subjects, 11 males and 2 females, completed the two experimental sessions. As for men, the mean age was 24.2 ± 3.3 years (range 19–32 years); mean weight was 76.3 ± 11.9 kg (range 65–105 kg), and mean height was 1.8 ± 0.2 m (range 1.7–1.9 m). The women were 23 and 25 years old, weighed 56 and 63 kg, and were 1.59 and 1.67 m tall, respectively. Globally, the subjects had an average onset of cannabis use at 17.5 ± 1.7 years (range 14–19 years), with a mean of 30 cannabis sessions in the last year and a mean of 0.90 joints by consumption. All subjects had a history of oral cannabis use at least once in their life, 53% of subjects had ingested hallucinogenic mushrooms on some occasion, and 38% had ever used cocaine, ecstasy, amphetamines, or MDMA. Regarding smoking, 46% of subjects were tobacco smokers and all had experience with alcohol intake, with a mean consumption of 1.19 alcoholic units/day. The thirteen subjects were administered 100 mL of the FM2 cannabis decoction containing 0.3 ± 0.12 mg THC, 1.2 ± 0.4 mg THCA-A, 0.7 ± 0.4 mg of CBD, and 4.4 ± 0.6 mg of CBDA. After wash out period, they were administered 0.45 mL of FM2 cannabis oil containing 1.0 ± 0.2 mg THC, 1.4 ± 0.3 mg THCA-A, 0.9 ± 0.2 mg of CBD and 2.8 ± 0.4 mg of CBDA. Significantly higher amounts of THC and CBD were observed in cannabis oil and significantly higher amounts of CBDA in cannabis decoction after preparation. We studied the disposition of CBD metabolites in subjects’ serum and urine samples but did not investigate their presence in oral fluid or sweat since we previously demonstrated that THC metabolites were not excreted in these latter biological matrices [12], and preliminary analyses for CBD metabolites in a couple of samples also confirmed the absence of these metabolites.

### 2.2. Concentration–Time Profiles and Pharmacokinetics of CBD Metabolites in Serum after Decoction and Oil Administration

**Figure 1:**
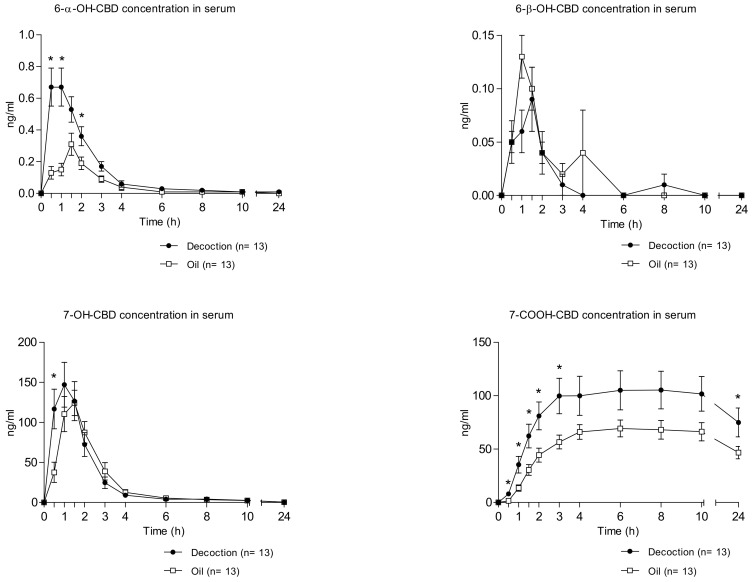
Mean time course of 6-alpha-hydroxycannabidiol (6-α-OH-CBD), 6-beta-hydroxycannabidiol (6-β -OH-CBD), 7-hydroxycannabidiol (7-OH-CBD), and cannabidiol-7-oic acid (7-COOH-CBD) concentrations in serum following the administration of cannabis decoction and oil (*n* = 13, mean values ± standard error). The time course of analytes’ concentrations was analyzed employing a two-way, repeated-measures ANOVA with formulation and time as factors. When treatment condition or the treatment condition × time interaction was statistically significant, a Student’s t-test for paired samples was performed. The symbol * shows significant differences between decoction and oil concentration.

Figure 1 shows the mean time course of 6-α-OH-CBD, 6-β-OH-CBD, 7-OH-CBD, and 7-COOH-CBD concentrations in serum following the administration of decoction and oil.

It can be noted that 6-β-OH-CBD showed the lowest serum concentrations among the detected metabolites, with a peak concentration 1.5 h after decoction intake comparable to the one measured 1 h after oil intake (0.12 ± 0.08 and 0.17 ± 0.11 ng/mL, respectively). Consequently, the area under the concentration–time curve at 10 and 24 h (AUC_0–10h_ and the AUC_0–24h_) of this metabolite were comparable following the administration of both formulations (Table 1). Moreover, 7-OH-CBD presented a similar time course (C_max_, AUC_0–10h_, AUC_0–24h_ and elimination profiles) in serum after both decoction and oil consumption; but differently from 6-β-OH-CBD, it was the CBD metabolite with the highest peak concentration in serum (C_max_ 159.93 ± 101.75 for decoction and 151.45 ± 58.81 for oil) (Table 1).

Conversely, 6-α-OH CBD and 7-COOH CBD showed different serum pharmacokinetic profiles for the two investigated cannabis preparations (Table 1). Although T_max_ was not statistically different after decoction and oil intake, significant differences were observed in mean peak concentrations (6-α-OH CBD C_max_ 0.80 ± 0.41 ng/mL for decoction and 0.42 ± 0.18 ng/mL for oil and 7-COOH CBD C_max_ 118.03 ± 64.94 for decoction and 74.73 ± 31.84 for oil) and in AUCs of both metabolites. Finally, it is worth noting that complete elimination of 7-COOH-CBD was not accomplished during the 24 h time of collection.

### 2.3. Urinary Excretion of CBD Metabolites after Decoction and Oil Administration

**Figure 2:**
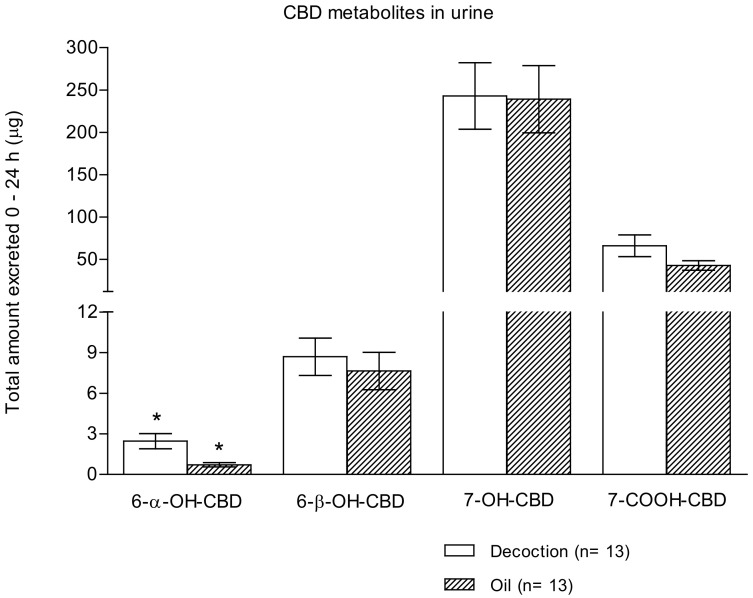
Total amount of 6-α-OH-CBD, 6-β-OH-CBD, 7-OH-CBD, and 7-COOH-CBD excreted in 24 h urine samples following the administration of cannabis decoction and oil (*n* = 13; mean ± standard error). The symbol * shows significant differences between decoction and oil preparations values.

Following the administration of both herbal preparations, all the investigated metabolites were excreted in urine, although in significantly different total amounts (Figure 2).

At the first collection time interval (2 h after the start of experiment), all the investigated metabolites could be measured in urine samples. Apart from 6-α-OH-CBD, which had significantly lower excretion after oil administration (*p* < 0.001), all the other metabolites were similarly eliminated in 24 h urine samples both after decoction and oil administration.

The most excreted CBD metabolite was 7-OH-CBD (mean ± SD: 242.9 ± 141.35 μg in the case of decoction and 239.1 ± 143.1 μg in the case of oil). Furthermore, 7-COOH-CBD was the second most prevalent metabolite, about four time less concentrated than the previous one (65.9 ± 46.2 μg and 42.7 ± 20.3 μg after decoction and oil consumption, respectively). Additionally, 6-β-OH-CBD was detected in urine samples at a concentration 30 times lower than that of the hydroxy metabolite (8.7 ± 4.9 μg for decoction vs. 7.6 ± 5.0 μg for oil), and finally, 6-α-OH-CBD was excreted in urine with concentrations two orders of magnitude less (2.5 ± 2.0 μg after decoction intake and 0.7 ± 0.6 μg after oil intake).

## 3. Discussion

To the best of our knowledge, this is the first study on CBD metabolites’ disposition in serum and urine samples of adult healthy volunteers after controlled administration of medical cannabis decoction and oil.

Analyzing the same samples, we recently reported the kinetics of THC, CBD, their acidic precursors, and THC metabolites. In that vein, we observed that even if cannabis oil contained a significantly higher amount of THC, its bioavailability and that of its metabolites was similar in both herbal preparations. Conversely, the THC acidic precursor was equivalently present in both preparations and correspondently absorbed in serum. Whereas oil contained a significantly higher amount of CBD and a lower amount of CBDA, absorption was significantly higher after decoction intake for both compounds. THC metabolites showed a later onset with respect to parent drug in the absorption phase and a slower decrease to baseline. Whereas 11-OH-THC presented similar disposition after administration of the two herbal preparations, THC-COOH and THC-COOH Gluc absorption was greater after decoction administration, in agreement with what was reported for the parent cannabinoid [12].

Considering previously observed different CBD (1.2 ± 0.6 ng/mL after decoction administration vs. 0.4 ± 0.3 ng/mL after oil administration peaking at a mean of 2 h peak time) and CBDA (91.4 ± 40.5 ng/mL after decoction administration vs. 28.3 ± 17.4 ng/mL after oil administration at a mean of 1 h peak time) bioavailability between the two cannabis reparations at the same dose, CBD metabolites’ disposition was expected to vary following decoction and oil administration. In reality, a different disposition was observed in serum only in the case of the least concentrated CBD metabolite, 6-α-OH-CBD, and the most concentrated one, 7-COOH-CBD, whose biosynthesis was significantly higher for decoction than for oil. In the case of 6-α-OH-CBD, a higher formation following decoction intake was confirmed by a greater urinary excretion, which was not the case for 7-COOH-CBD, which showed a trend toward higher elimination after decoction consumption.

CBD metabolites’ disposition following medical cannabis administration confirms that CBD is prevalently metabolized to the 7-carboxy metabolite, as already found by previous studies [13,14]. This metabolite was the most concentrated in serum but not the most excreted in urine, since at 24 h post experiment start, it was not yet totally eliminated from serum. Furthermore, taking into account the previously reported concentrations of CBD/CBDA [9], the current results demonstrate that concentrations of 7-COOH-CBD and 7-OH-CBD are more than 10 or 3 times higher than that of the parent substance, respectively.

There are few previous investigations on the pharmacokinetics of CBD metabolites in humans.

One early study established that after the administration of a of 20 mg [3H]CBD dose to healthy volunteers by intravenous injection, 7-COOH-CBD was the most abundant metabolite in the plasma, while 7-OH-CBD was only a minor metabolite (the compounds were referred to as 11-carboxy-CBD and 11-hydroxy-CBD, respectively) [15].

More recently, the pharmacokinetic properties of CBD and some related metabolites were assessed after single- and multiple-dose administration of a highly purified pharmaceutical CBD preparation [14]. A single dose of 1500, 3000, 4500, or 6000 mg was orally administered to four groups of healthy volunteers and CBD, 7-COOH-CBD, and 6-OH-CBD were quantified in plasma samples. The reported metabolic profile was similar to the one here presented, with 7-COOH-CBD being the most prevalent serum metabolite, followed by 7-OH-CBD and 6-OH-CBD.

More than what they have shown, we measured 6-OH metabolite isomers, which displayed different concentration values both in serum and in urine. The 6-β-OH-CBD was the most prevalent in both investigated matrices after the administration of each different preparation.

The above-cited study indicated also that metabolites’ T_max_ in serum was around 4.6 h, independent of the administered dose. Conversely, T_max_ observed in our study was shorter (1.5 h) for all the metabolites apart from 7-COOH-CBD (8 h), without any difference between the two administered preparations. Although that and our investigation reached similar results concerning CBD metabolites disposition, the divergent T_max_ values might be due to the presence of THC in the herbal decoction and the oil, while it was not present in the pharmaceutical CBD preparation [14]. Finally, in our study, a significant interindividual variability in CBD metabolites’ concentration both in serum and urine was observed among the enrolled healthy subjects selected, in agreement of what previously reported in other studies on individuals treated with cannabis herbal preparations [9,11,12]. As a matter of fact, the most important limitations are indeed the inclusion of healthy volunteers, predominantly males, the very small number of tested individuals, and the low administered doses.

## 4. Materials and Method

### 4.1. Subjects’ Enrolment

Twelve healthy males and two healthy females, were recruited for the two sessions of the study by “word of mouth” and the database of volunteers who had participated in previous studies at Clinical Pharmacology Unit, Hospital Universitari Germans Trias i Pujol, Badalona, Spain. Participants were informed of the characteristics of the study and signed an informed consent form. Eligibility criteria included social or recreational cannabis use in the last 12 months (from weekly to monthly to use, without criteria of cannabis use disorder) and oral cannabis use at least once in participants’ life (e.g., cakes, cookies, oil, and/or infusion). Exclusion criteria were an history of any serious medical diseases or chronic medication use, any or psychopathological condition including substance use disorder (except for nicotine, according to the Diagnosis and Statistical Criteria for Mental Disorders, DSM-5), chronic medication use, and severe adverse reactions associated with cannabis use, pregnancy, and lactation.

The participants underwent a general medical examination, including blood and urine analysis and a 12-lead electrocardiogram (ECG) within three weeks prior to inclusion and were given time to familiarize with study procedures and questionnaires. The study protocol was approved by the local Human Research Ethics Committee (CEIC-HGTiP, Badalona, Spain), and the study was conducted in accordance with the Declaration of Helsinki and Spanish laws concerning clinical research.

### 4.2. Cannabis Decoction and Oil Preparation

With the authorization of the Italian and Spanish Medicines Agencies, the Italian Military Pharmaceutical Chemical Factory in Florence provided us with a standardized medicinal cannabis flower cup extract called FM2 that contains 5.9% THC and 8.4% CBD. The preparation of the decoction and the oil was performed as previously reported [9,13]. A decoction volume of 1 mL and three drops of oil were saved for later analysis in order to verify the exact amount of THC and CBD administered.

### 4.3. Study Design

The study was open label, not randomized, and single blinded, since participants were aware of the cannabis preparation but not of the administered doses. The study included two separate experimental sessions, the first one for decoction administration and second one for oil administration, separated by at least 3 weeks. Further details were previously reported [9]. After an overnight fast, the subjects were admitted to the Clinical Research Unit (UPIC) at 07:45 a.m. Upon arrival of the subjects, they were asked about any substance/drug or event that could affect their participation in the study. The subjects had previously been instructed not to consume psychoactive drugs for at least the seven days prior to the study or products with caffeine and alcohol for the previous 24 and 48 h, respectively.

Before starting each the of the two experimental sessions, a urine sample was collected for drug detection (Instant-View^®^, Multipanel 10 Test Drug Screen, Alfa Scientific Designs Inc., Poway, CA, USA), and analysis of alcohol was performed in exhaled air (Dräger alcotest 5820, Dräger, Germany). If either of these two tests was positive, the subjects were excluded from the experimental session. The subjects remained sitting in bed in the Hospital’s Clinical Research Unit (UPIC), throughout the session. The last cigarette allowed was 2 h before starting the session, smoking being prohibited during the course of the study. Subjects were placed on an intravenous catheter for the collection of blood samples, and sweat patches were applied on the back. The administration of decoction (100 mL) or oil (15 drops, 0.45 mL) was carried out between 08:15 and 08:30 h. After the herbal preparations’ administration, the mouth was washed twice with water to avoid oral contamination in oral fluid collection. Subjects received breakfast, lunch, and a snack two, five, and eight hours respectively, after administration. The duration of the experimental sessions was 10 h, and subjects returned to the center at 24 h after administration. Other variables related to pharmacological effects were evaluated and reported in a previous publication [9].

### 4.4. Biological Samples’ Collection

Serum (2 mL) from whole-blood centrifugation was collected from 15 min prior to administration (baseline, 0 h) and 0.5, 1, 1.5, 2, 3, 4, 6, 8, 10, and 24 h after decoction or oil administration. Urine was collected 15 min prior to administration (zero time) and then between 0–2, 2–4, 6–8, 8–10, and 10–24 h intervals after administration.

### 4.5. Determination of Cannabinoids in Herbal Preparations and CDB Metabolites in Serum and Urine Samples

THC, CBD, THCA-A, and CBDA were quantified in herbal preparations by ultra-high-performance liquid chromatography–tandem mass spectrometry (UHPLC-MS/MS) equipped with an electrospray ionization source operating in positive-ion mode as previously reported [15]. With respect to 6-α-OH-CBD, 6-β-OH-CBD, 7-OH-CBD, and 7-COOH-CBD, 99% pure standards were purchased from Dalton Research Molecules (Toronto, ON, Canada), and a specific UHPLC-MS-MS assay has been recently developed and validated with limits of quantification ranging from 0.05 to 0.1 ng/mL. with average inter-/intra-day accuracy and precision <15% [16].

### 4.6. Statistical Analysis

Maximum concentration (C_max_), time needed to reach maximum concentrations (T_max_), area under the concentration–time curve at 10 and 24 h (AUC_0–10h_, AUC_0–24h_), elimination half-life (t_1/2_), and elimination constant (K_e_, calculated with at least three sample points) for CBD metabolites in serum were determined using Pharmacokinetic Functions for Microsoft Excel (Joel Usansky, Atul Desai, and Diane Tang-Liu, Department of Pharmacokinetics and Drug Metabolism, Allergan, Irvine, CA, USA at https://www.coursehero.com/file/30859156/pkfdoc/).

A Student’s *t*-test for paired samples was performed to assess potential differences between pharmacokinetic parameters in the decoction and the oil, since a Kolmogorov–Smirnov test showed a normal parameter distribution. A nonparametric Wilcoxon signed-rank test for paired samples was instead applied for T_max_, not a continuous variable and non-normally distributed.

The time course of analytes concentrations was analyzed employing a two-way, repeated-measures ANOVA with formulation and time (0–10 h) as factors. When treatment condition or the treatment condition × time interaction was statistically significant, a Student’s *t*-test for paired samples was performed to compare analytes’ concentrations in the decoction and oil. All statistical tests were performed at each time point using the PASW Statistics 18.0 (SPSS Inc., Chicago, IL, USA). A value of *p* < 0.05 was considered statistically significant.

## 5. Conclusions

The present study aimed to characterize and compare the disposition of CBD metabolites in serum and urine after the controlled administration of medical cannabis decoction and oil. Notwithstanding the highlighted study limitations, the pharmacokinetic profile of the most representative CBD metabolites showed several differences between the decoction and the oil, in accordance with the different availability of CBD for the two formulations. Furthermore, the pharmacokinetic characterization of CBD metabolites is crucial for the clinical practice since cannabis herbal preparations are increasingly used for several pathological conditions.

## Figures and Tables

**Table 1:** Pharmacokinetic parameters of cannabidiol (CBD) metabolites in serum of healthy volunteers after administration of 100 mL cannabis decoction and 0.45 mL cannabis oil (*n* = 13; SD = standard deviation).

Parameters	Cannabis Decoction (Mean ± SD)	Cannabis Oil (Mean ± SD)	*p* Value
6-α-OH-CBD			
C_max_ (ng/mL)	0.80 ± 0.41	0.42 ± 0.18	**0.004**
T_max_ (h)	1.0 (0.5–2)	1.5 (0.5–3)	0.115
AUC_0–10h_ (ng/mL·h)	1.59 ± 0.85	0.68 ± 0.33	**0.005**
AUC_0–24h_ (ng/mL·h)	1.69 ± 0.84	0.78 ± 0.47	**0.006**
K_e_ (h^−1^)	0.39 ± 0.35	0.55 ± 0.34	0.421
t_1/2_ (h)	4.62 ± 5.37	2.37 ± 2.65	0.349
6-β-OH-CBD ^§^			
C_max_ (ng/mL)	0.12 ± 0.08	0.17 ± 0.11	0.067
T_max_ (hour)	1.5 (0.5–3)	1.0 (0.5–4)	1.000
AUC_0–10h_ (ng/mL·h)	0.16 ± 0.10	0.25 ± 0.29	0.223
AUC_0–24h_ (ng/mL·h)	0.17 ± 0.11	0.26 ± 0.29	0.217
7-OH-CBD			
C_max_ (ng/mL)	159.93 ± 101.75	151.45 ± 58.81	0.727
T_max_ (hour)	1.0 (0.5–2)	1.5 (1–2)	0.194
AUC_0–10h_ (ng/mL·h)	306.73 ± 204.52	280.00 ± 128.62	0.587
AUC_0–24h_ (ng/mL·h)	327.54 ± 217.36	299.37 ± 140.64	0.586
K_e_ (h^−1^)	0.21 ± 0.06	0.26 ± 0.09	0.170
t_1/2_ (h)	3.51 ± 0.94	2.89 ± 0.74	0.118
7-COOH-CBD ^§^			
C_max_ (ng/mL)	118.03 ± 64.94	74.73 ± 31.84	**0.031**
T_max_ (hour)	8.0 (3–24)	6.0 (4–24)	0.272
AUC_0–10h_ (ng/mL·h)	885.94 ± 531.41	552.89 ± 225.26	**0.036**
AUC_0–24h_ (ng/mL·h)	2122.73 ± 1257.15	1343.35 ± 569.39	**0.034**

C_max_, maximum concentration; T_max_, time needed to reach maximum concentration; AUC_0–10h_ and AUC_0–24h_, area under the concentration–time curve at 10 and 24 h; K_e_, elimination constant; t_1/2_, elimination half-life. ^§^ K_e_ and t_1/2_ could not be calculated since the number of decreasing points to calculate these parameters was insufficient (less than three). In bold *p* < 0.05.
